# Pharmacological Management for Pediatric Irritable Bowel Syndrome: A Review

**DOI:** 10.7759/cureus.49197

**Published:** 2023-11-21

**Authors:** Alaa S Alyasi, Mohammed A Altawili, Asmaa F Alabbadi, Asma Hussain A Hamdi, Amjad S Alshammery, Mohammed I Alfahad, Rahaf M Alamri, Talal R Alanazi, Maram Hafiz A Harbi, Alaa M Alajmi, Jehad M Alabdulrahim, Amani M Alalshaikh, Afnan M Hanbzazah

**Affiliations:** 1 Pediatrics and Neonatology, Maternal and Child Health Care Center, Tabuk, SAU; 2 General Practice, Al Aziziyah Primary Health Care Center, Tabuk, SAU; 3 Medicine, AlMaarefa University, Riyadh, SAU; 4 Family Medicine, University of Najran, Najran, SAU; 5 General Practice, King Salman Specialist Hospital, Hail, SAU; 6 General Practice, Ahad Rafidah General Hospital, Ahad Rafidah, SAU; 7 Medicine, King Abdulaziz University, Jeddah, SAU; 8 Medicine, Jouf University, Sakaka, SAU; 9 Medicine, Jazan University, Jizan, SAU; 10 General Practice, Primary Health Centers - Dammam Health Network, Dammam, SAU; 11 Medicine, Qassim University, Buraydah, SAU; 12 Pediatrics, Iman General Hospital, Riyadh, SAU; 13 Pediatrics, King Abdulaziz Hospital, Jeddah, SAU

**Keywords:** pediatrics, rome criteria, manning criteria, pharmacology, irritable bowel syndrome

## Abstract

Irritable bowel syndrome is a multifactorial disease with chronic symptoms that interfere with the quality of life of patients. It represents one of the most common causes of functional abdominal pain in the pediatric population. Various theses with little evidence tried to explain the pathophysiology of the disease. Neurological origin was one of the theories explaining the disease, either by the disturbance of neurotransmitters like dopamine, noradrenaline, and serotonin, which have some evidence of their relation to GI tract functions. Other factors like bio-psycho-social factors that affect the pediatric population are represented in bullying, unrealistic academic expectations from the parents, continuous educational stress, and difficult relationships with peers. Other factors may be genetic abnormalities of the receptors or visceral hypersensitivity. Treatment strategies for the disease varied from physical activity like yoga to a diet like a low-FODMAP diet. Pharmacological treatment of the disease targets the presenting symptoms, represented by antispasmodic drugs treating abdominal pain/discomfort, antipsychotics that regulate the disturbance in the brain-gut axis, and other drugs targeting diarrhea or constipation that present with the patient according to the type of IBS and the condition of the patient.

## Introduction and background

Irritable bowel syndrome (IBS) is one of the common functional GI disorders, characterized by various symptoms, mostly chronic abdominal pain/discomfort, bloating, flatulence, constipation, diarrhea, and alternating bowel habits. According to ROME III criteria, IBS is a clinical syndrome characterized by recurrent abdominal pain/discomfort at least three days monthly in the previous three months associated with more than two of the following: improvement with defecation, onset associated with a change in stool frequency and form, and the symptoms must have started for at least six months before [1,2]. Based on the stool characteristics, we could categorize IBS into (1) IBS with constipation (IBS-C) characterized by more than 25% hard/lumpy stools and less than 25% loose or watery stools; (2) IBS with diarrhea (IBS-D) characterized by more than 25% loose or watery stools and less than 25% hard/lumpy stools; and (3) IBS with mixed stool pattern (IBS-M) characterized by more than 25% hard/lumpy stools and more than 25% loose or watery stools [1]. It has a pediatric population prevalence of nearly 8.8% according to the region [3]. IBS has several triggering factors like altered GI motility, visceral hypersensitivity, intestinal microbiota imbalance, altered brain-gut axis, digestive tract inflammation, and psychological factors that appear to determine the occurrence and progression of IBS [4,5].

Since IBS is a multifactorial disease and the variability of the presenting symptoms, the treatment options target only the presenting symptoms. Many treatment options have developed to either relieve the abdominal pain/discomfort, constipation/diarrhea, or the accompanied psychological symptoms. Some treatment strategies depend on patients' diets like diets low in fermentable oligosaccharides, disaccharides, monosaccharides, and polyols (FODMAP), gluten-free diets (GFD), and starch- and sucrose-reduced diets (SSRD) [6]. Other treatment strategies depend on pharmacological therapy to target specific mechanisms such as inflammatory, immune, and neuroendocrine mechanisms that contribute to visceral hypersensitivity and chronic inflammation of the small intestine and colon, as well as altered intestinal permeability [7]. The brain-gut axis is a target to be modulated in IBS, mainly by virtue of neurotransmitter levels. Pain modulation, GI dysmotility, and alterations in neurotransmitters and their receptors appear to play an essential role in the development of IBS [8]. In post-infection IBS, probiotics generate some metabolites that can inhibit pathogen growth [9]. Certain probiotic strains can also assist with enzyme production to improve the digestion of lactose and facilitate bile salt deconjugation to modulate blood lipids [10]. Some important symptoms, such as abdominal pain, were managed by antispasmodics by relaxing abdominal smooth muscles and organizing intestinal motility. Consequently, this review will discuss the pathophysiology, diagnosis, and pharmacological management of pediatric IBS.

## Review

Methods

We have conducted a systematic search about the available pharmacological therapy for IBS among children using three searchable databases: Web of Science, PubMed, and Scopus, up to August 2023. The search terms used were: “irritable bowel syndrome,” “irritable colon,” “IBS,” ”pediatric,” ”pharmacological therapy,” etc. The search process was not limited to English. Also, we searched the literature manually to gather information about the following points: (1) factors related to IBS incidence; (2) patients older than four years; (3) ROME I, II, III, or IV diagnostic criteria; (4) pharmacological treatment of abdominal pain, anxiety, etc.; and (5) results such as reduction in IBS symptoms, improvement in quality of life, and stool regularity/frequency. This review article was conducted between 1 April 2023 and 19 August 2023.

Pathophysiology

Physiological symptoms of IBS such as gastric motility and altered visceral sensitivity showed a close relationship to the neurotransmitters of patients [11]. Several studies have clarified the role of (5-hydroxytryptamine (5-HT)) neurotransmitter on the gut's physiological functions. 5-HT increases when the intestinal tract is stimulated and binds to 5-HT_3_ receptors in the exogenous primary afferent nerve endings, increasing the enteric nervous system and visceral afferent nerve sensitivity which causes discomfort, abdominal pain, and diarrhea [12]. According to Fu et al. (2019), expression of the 5-HT and 5-HT_3_ receptors in the intestinal mucosa was significantly higher in the IBS-D group than in the healthy control group indicating an impaired 5-HT system in IBS patients [13]. Other neurotransmitters like gamma-aminobutyric acid, histamine, and dopamine have shown a significant relationship between their disturbance and the physiological symptoms of different types of IBS. Their pivotal role in normal functions, such as acid secretion, gastric emptying, intestinal motility, and pain perception, increases the permeability of the intestinal mucosa and affects mucosal ion secretion [12]. IBS showed a relevant association with the neuroendocrine system. The neuropeptide Y is present in the central and peripheral nervous systems, as well as in the digestive tract. There is a positive correlation between serum cortisol and neuropeptide Y in healthy individuals, which are used as markers for the activation of the hypothalamic-pituitary-adrenal axis and the sympathetic fibers of the autonomic nervous system (ANS) axis. Neuropeptide Y is also a modulator between the ANS and mast cells, linking the psychological state to the symptoms of IBS [14]. IBS patients reported low-intensity pain when their gut was stimulated contrary to non-IBS patients which suggests that the cause may be neurological [15,16].

Furthermore, there are some risk factors for irritable bowel disease like GI infection as 10% of patients with GI infections develop irritable bowel disease, family history of IBS, and some life events, like trauma and psychological abuse, as well as personality traits like neuroticism and pessimism, and psychological distress caused by factors like life stress, anxiety, and depression [17].

Diagnosis

The diagnosis of IBS in children relies on the symptoms they report, and no specific laboratory test is used to confirm it. The most crucial aspect of establishing a diagnosis of IBS in children involves a thorough history and physical examination [18]. An IBS diagnosis is confirmed after excluding any sign of an inflammatory, anatomic, metabolic, or neoplastic disorder that explains these symptoms. IBS affects the psychological status of children or their quality of life. IBS sufferers require frequent hospital visits, which are expensive [19].

The Manning criteria, which is the first diagnostic criteria for IBS, was established in 1978 based on symptoms that were more frequent in patients with IBS than other organic diseases. The criteria included four main symptoms: pain, loose stool, increased abdominal movement, and abdominal distension. The stool becomes loose at the beginning of the pain. Then, abdominal movement increases, and pain relief occurs. Finally, patients suffer abdominal distension. Also, the sensation of incomplete evacuation and mucus in stool is a more prevalent symptom in IBS patients (also included in the Manning criteria). When two of the four main symptoms were present, the criteria had a sensitivity of 91% and a specificity of 70%. When two of all six symptoms were present, the sensitivity ranged from 84% to 94% and the specificity was 55%. Finally, when ≥3 of the six symptoms were present, the sensitivity ranged from 63% to 90% and the specificity from 70% to 93%. Despite these data, this criteria does not differentiate between IBS with constipation and IBS with diarrhea [2].

In 1984, Kruis et al. reported symptoms similar to those used to define IBS: abdominal pain, bloating, and altered bowel function. Unlike the Manning criteria, the Kruis criteria placed greater importance on symptom duration and suggested two years. Moreover, the Kruis criteria stressed the importance of considering warning signs and excluding organic disease through a combination of a normal physical examination, basic laboratory studies, such as a complete blood count and erythrocyte sedimentation rate, and endoscopy. However, these criteria ultimately proved too burdensome for practical use and have since fallen out of favor [18].

In 1992, new criteria were established for the diagnosis of IBS called ROME I depending on the symptom scheme that happened in IBS. However, the criteria did not differentiate between abdominal bloating and abdominal pain, which are cardinal symptoms of many IBS patients. A study evaluated the diagnostic accuracy of the ROME I criteria in 339 IBS patients, with a reported sensitivity of 85% and a specificity of 71% [20]. Several years later, ROME criteria were updated based on feedback from clinicians to ROME II. ROME II defines IBS patients by 12 weeks of abdominal pain at least, which was relieved with the defection, and change in stool frequency and appearance. However, patients were not categorized into specific subtypes based on bowel habits at that time [21].

In 2006, the ROME II criteria were updated to the ROME III criteria to classify IBS based on stool consistency. There are three subtypes of IBS: constipation-predominant (IBSC), diarrhea-predominant (IBS-D), and mixed type (IBS-M), based on the dominant type of stool during pain episodes. The ROME III criteria are the most commonly used clinical criteria for diagnosing IBS in children. If a child complains of abdominal pain relieved by defecation, change in stool frequency or appearance, and there is no sign of any inflammatory, anatomic, metabolic, or neoplastic action that explains the symptoms, the child likely has IBS [11]. A validation study conducted by Ford et al. on patients with IBS symptoms who underwent colonoscopy found that the ROME III criteria had a sensitivity of 68.8% and a specificity of 79.5% [22].

ROME IV classifies IBS as a functional GI disorder characterized by recurrent abdominal discomfort that occurs with defecation or a change in bowel habits. Persistently disrupted bowel habits (constipation, diarrhea, or a combination of both), abdominal bloating, and distension are common. The patient must have experienced these symptoms for at least six months before diagnosis and for at least three months after [2]. The ROME IV criteria differ from the ROME III criteria in that the average frequency of abdominal pain was changed from three days per month to one day per week. Although this modification appears minor, it is the result of studying large population data to increase the sensitivity and specificity of the ROME criteria [23]. Finally, IBS is characterized by predominant changes in bowel habits on days with irregular bowel movements.

Pharmacological treatment

As IBS is a multifactorial disease without any defined way of management, most of the management strategies are based on symptomatic treatment. Thus, we will discuss several pharmacological therapies that have effects on the pediatric population.

Oral Psyllium

Oral psyllium is one of the drugs for the treatment of irritable bowel disease, which makes the stool bulky and soft due to its water-holding capacity. Oral psyllium also has prebiotic and immunomodulatory effects [24,25].

Menon et al. (2023) discussed the efficacy of oral psyllium in the treatment of irritable bowel disease according to the irritable bowel disease severity scoring scale (IBS-SSS). The baseline mean of IBS-SSS in the psyllium group was 265.976±84.60 and in the placebo group was 289.35±68.41, but after the treatment, the mean of IBS-SSS in the psyllium group was 102.31±94.77 (P=0.048) and in the placebo group was 225.161±106.54 (P=0.00) with a relative risk of 0.64 (95% CI; 0.47, 0.84: P=0.0017) and a relative risk reduction (1 − RR) of 0.36 (36%) [24]. Regarding pain frequency, Shulman et al. (2017) compared psyllium against placebo and found the mean of pain episodes was approximately similar in the two groups at baseline in the treatment group (16.68±1.657) and the placebo group (15.02±1.9), but after the treatment, there was a significant decrease in pain frequency in the psyllium group (8.4±1.893) compared with the placebo group (11.24±1.65) [26]. According to these data, oral psyllium is effective in the treatment of patients with irritable bowel disease. Consequently, oral psyllium is effective in reducing the severity and pain frequency in IBS patients.

VSL#3

VSL#3 consists of live, freeze-dried lactic acid bacteria with a total concentration of 450 billion lactic acid bacteria per sachet, including eight different strains: *Bifidobacterium breve*, *Bifidobacterium longum*, *Bifidobacterium infantis*, *Lactobacillus acidophilus*, *Lactobacillus plantarum*, *Lactobacillus casei*, *Lactobacillus bulgaricus*, and *Streptococcus thermophilus* [27].

Guandalini et al. (2010) compared VSL#3 versus placebo in the subject's global relief of symptoms at baseline in the mixture group (4.1±0.22) and the placebo group (4.1±1.7). After six weeks of treatment, the score became in the mixture group (2.2±0.29) and the placebo group (3.06±0.35) [27]. There is a significant reduction in the score in the VSL#3 (probiotic mixture) group. Abdominal pain which is the most significant symptom of IBS is also affected by VSL#3. In Guandalini et al. (2010), the baseline abdominal pain score of the VSL#3 group was 2.12±0.2 and the placebo group was 1.9±0.18 [27]. After six weeks of receiving VSL#3, the mean abdominal pain score became 1.09±0.13 in the VSL#3 group and 1.46± 0.13 in the placebo group [27]. Additionally, Guandalini et al. (2010) found a reduction in abdominal bloating scores in the VSL#3 group. The baseline abdominal bloating score of the VSL#3 group was 2.4±0.18 versus 2.1±0.2 in the placebo group. After six weeks of receiving VSL#3, the mean abdominal bloating score in the VSL#3 group became 1.05±0.14 versus 1.6±0.2 in the placebo group. Therefore, VSL#3 (probiotic mixture) shows a significant effect in the treatment of IBS [27].

Bacillus coagulans Unique IS2

*Bacillus coagulans* Unique IS2 is one of the probiotics used in the treatment of IBS. Sudha et al. (2018) compared *Bacillus coagulans* Unique IS2 against placebo. The mean pain score in the treatment group was 3.8±0.55 versus 3.7±0.49 in the placebo group. After eight weeks of treatment, it increased in the treatment group (7.62±0.98) and in the placebo group (4.23±1.40) (P<0.001), and this increase in the numeric rating scale refers to a reduction of pain intensity [28]. Sudha et al. (2018) used an 11-point Likert scale to estimate pain intensity, where 0 represents no improvement and 10 is the highest improvement [28].

Lactobacillus rhamnosus Strain GG

*Lactobacillus rhamnosus* strain GG (LGG) is one of the probiotics that is effective in decreasing the severity of pain in IBS patients. In Francavilla et al. (2010), the severity of pain in the LGG and placebo groups was similar (4.3±1.8), but after 12 weeks of treatment, the pain severity decreased in the LGG group to 2.3±1.3 and in the placebo group to 3.4±2.1. From these data, LGG is effective in reducing the severity of pain in IBS patients [29]. For the abdominal pain and abdominal muscle spasms, antispasmodics are being administered to relieve the symptoms.

Trimebutine

Trimebutine maleate is an opioid agonist that acts on the peripheral delta, mu, kappa, and delta opiate receptors. It works by modulating the release of peptides such as vasoactive intestinal peptides, gastrin, and glucagon. It also induces the release of motilin which stimulates gastric and small intestine motility. Karabulut et al. (2013) conducted a study on 78 children with IBS and found a significant benefit of trimebutine maleate compared with a non-medicated group. Children who received trimebutine achieved more clinical recovery compared to children who had spontaneous recovery (P<0.0001). Although no side effects were reported in the study [30].

Peppermint Oil

It is one of the essential oils extracted by steam distillation from the fresh leaves of peppermint that has been used to treat abdominal discomfort [31]. The active ingredient of peppermint oil l-menthol has multiple mechanisms for caring for functional abdominal pain. Peppermint oil works as a smooth-muscle calcium channel blocker [32]. The amplitude and rate of the contractions have decreased significantly accompanied by an increase in the length of phases I and II but a shorter phase III in the migrating motor complex [33].

Drotaverine

Drotaverine is a selective inhibitor of phosphodiesterase isoenzyme IV, which is effective in smooth muscle spasms and motility disorders as it acts as a smooth muscle relaxant without anticholinergic effects [34,35]. Narang et al. (2015) compared drotaverine versus placebo and found that the baseline abdominal pain episodes were approximately similar in the two groups, but after four weeks, the abdominal pain episodes were 10.3±14 in the drotaverine group and 21.6±32.4 (P=0.015) in the placebo group, showing that drotaverine is effective in the treatment of IBS in children [34]. As IBS has a neurological basis too, some drugs are used to alleviate this cause.

Citalopram

Citalopram is a selective serotonin reuptake inhibitor medication that can improve symptoms of IBS by increasing central serotonin as it reduces abdominal motility in patients with IBS-D and increases motility in IBS-C [36]. Roohafza et al. (2014) [37] compared citalopram versus placebo and found that in the baseline, the pain score was approximately similar in the citalopram group (3.8±0.8) and in the placebo group (3.6±0.8), but after 12 weeks, the pain score became 1.84±1.56 in the treatment group and 1.44±1.5 in the placebo group. There is no difference between the two groups. More RCTs are needed with large populations to evaluate the effectiveness of citalopram in children with IBS.

Amitriptyline

Amitriptyline is a tricyclic antidepressant. The FDA approved it for functional abdominal pain in adults, and it is approved for depression in children. In abdominal pain, amitriptyline decreases the pain threshold through its antinociceptive action through peripheral and central pathways [38-40]. It works on both pain score and quality of life. Seetharaman et al. (2022) compared amitriptyline and placebo and found that their mean pain score pre-treatment was 16.7±7.3 and 13.9±5.5 (P<0.0001), respectively, but post-treatment was 6.1±5.9 and 11.5±5.9 (P<0.0001), respectively [38]. In Saps et al. (2009), following the administration of amitriptyline or placebo to the children, 59% in the amitriptyline group reported feeling well, with 4% feeling worse. In contrast, in the placebo group, 53% felt better, while 2% felt worse. In their analysis, there was no significant difference between amitriptyline and placebo [39].

In Saps et al. (2009), the duration of treatment was four weeks shorter than in Seetharaman et al. (2022) [38], with a duration of 12 weeks. Also, the number of participants in Seetharaman et al. (2022) [38] was 168, more than the 90 patients in Saps et al. (2009) [39]. According to these data, amitriptyline can reduce pain in patients with irritable bowel disease. Quality of life is an important item affected by IBS as it causes some psychological disorders like stress, anxiety, depression, and somatization [41]. In Seetharaman et al. (2022) [38], the baseline mean quality of life score in the amitriptyline group was 2.7±1.9 versus 2.6±1.7 in the placebo group. However, post-treatment, there was a significant improvement in the quality of life score in the amitriptyline group (2.3±1.6), but in the placebo group, it became 0.9±1.6.

Tegaserod

Tegaserod is a selective serotonin receptor agonist that activates 5-HT4 receptors in the gut wall, leading to increased stool frequency, improved stool consistency, stimulation of the peristaltic reflex and intestinal secretions, and a reduction of visceral sensitivity [42]. Liem et al. (2008) evaluated the efficacy of tegaserod in children with functional abdominal disorders. After treatment with tegaserod, abdominal pain was moderate in 64% of the patients [43].

Domperidone

Domperidone is a butyrophenone derivative with anti-dopaminergic acts on the dopamine 2 receptors in the peripheral nervous system. It stimulates antral duodenal contractions, enhances coordinated peristalsis throughout the pylorus, and speeds up gastric emptying. Studies have demonstrated its effectiveness in improving the emptying of both solid and liquid foods in both healthy individuals and patients with delayed gastric emptying [44].

In the study by Karunanayake et al. (2018), the baseline pain severity was found to be similar in both the domperidone and placebo groups, with values of approximately 60.3±15.5 and 56.9±17.1, respectively. Although the difference in pain severity was not statistically significant (P>0.05), after eight weeks of treatment, there was a reduction in pain severity to 54.1±35.8 in the domperidone group and 21.6±32 and 29.7±50.2 in the placebo group, respectively. Additionally, the mean gastric emptying rate decreased from 46.6±12.2 in the domperidone group and 44.7±17.4 in the placebo group at baseline to 14.8±7.6 in the domperidone group and 7.4±11.2 in the placebo group, respectively, after eight weeks of treatment. These findings suggest that domperidone is effective in treating IBS in children [45].

Authors' personal view on preferred pediatric practices

Due to the insufficient evidence of treatment that will manage all children with IBS, it is preferred to start with a focused history of presenting symptoms that target potential causes that cause the condition, like the family, social, and educational history of the child, and consider a history of GI infections or any diseases that would trigger any of the factors discussed. Additionally, it is important to search for any possible red flags, especially in cases meeting the ROME III criteria.

Conducting a comprehensive physical examination and assessing growth, development, puberty, and the child’s mental status are integral. Due to the wide range of differential diagnoses of IBS symptoms, we will have to perform some elementary investigations to exclude organic causes. Our practice is to conduct a complete blood count, liver and renal function tests, inflammatory marker analysis, amylase assessment, celiac screening, and, in cases of diarrhea, stool culture and evaluation for stool-reducing substances. Some other special investigations, such as ultrasound scans of the abdomen, MRIs, GI endoscopies, etc., are only performed if we suspect any organic pathology and the test is appropriate.

Upon a positive diagnosis of IBS based on the ROME III criteria, most children will show promising outcomes with just counseling, education about IBS, and personalized management of pain, stress, and diet. We should explain to the parents that all the previous investigations done so far have yielded negative results, indicating nothing serious regarding these symptoms, thereby providing reassurance.

We will need to apply more specific strategies in cases with more severe disabling symptoms to achieve a better quality of life. Due to the complexity of the psycho-pathophysiology of IBS, there was no evidence of one strategy that could relieve all symptoms. Thus, the common approaches depend on symptom-targeted therapy.

Dietician counseling is needed to observe and follow the diet of the child. Many diets have been developed to suit each type of IBS and each patient. A high-fiber diet helps with IBS-C, while a low-fiber diet improves IBS-D, but it still varies from one patient to another according to their food tolerability and condition, as a lactose-free diet may be useful in cases of dairy intolerance.

Some bio-psycho-social approaches can help avoid the triggering symptoms of IBS or stop making them worse, like educating the family about IBS and addressing emotional or environmental issues. The pressure to meet parenting expectations continuously may lead to stress, necessitating counseling for support. Other social triggering factors, such as bullying at school, difficulties in relationships with parents or peers, unrealistic academic expectations, etc., should be explored more. More promising results could be achieved by an integrated team of pediatric gastroenterologists, dieticians, psychologists, social workers, and educators.

However, there is not sufficient evidence for the pharmacological treatments. Some drugs could alleviate the symptoms, like peppermint oil, which works as a smooth muscle relaxant, and trimebutine, which could help with abdominal pain or spasms. For severe psychiatric patients or patients with severe symptoms, we can use mood stabilizers. Eluxadoline on a required basis is useful in children with IBS-D. Antibiotics are needed in cases of small intestinal bacterial overgrowth or giardiasis.

Implications for future research

There is still little evidence to understand the pathophysiology of IBS in children. Long-term studies are required to investigate the effect of diet modification on managing IBS with a sufficient follow-up period. More studies should be implicated in biopsychosocial approaches. Comparison research should determine the superiority of ROME III and IV in the diagnosis of IBS. In difficult cases, a multi-disciplinary team approach is needed. Financial difficulties that a family may be facing are also worth exploring and addressing in future research.

## Conclusions

There is not much evidence regarding specific pharmacological treatment that could be generalized over the whole pediatric population, either because of the multifactorial nature of the disease or because the current studies were just targeting the adult population. Till now, the treatment of the condition has been based on each case circumstance. The current studies could recommend specific drugs for each symptom. However, further RCTs are recommended to provide a higher level of evidence of the condition and also other studies that discuss the factors that could just affect the pediatric population.

## References

[1] Halland M, Saito YA (2015). Irritable bowel syndrome: new and emerging treatments. BMJ.

[2] Lacy BE, Patel NK (2017). Rome criteria and a diagnostic approach to irritable bowel syndrome. J Clin Med.

[3] Korterink JJ, Diederen K, Benninga MA, Tabbers MM (2015). Epidemiology of pediatric functional abdominal pain disorders: a meta-analysis. PLoS One.

[4] Hyams JS, Di Lorenzo C, Saps M, Shulman RJ, Staiano A, van Tilburg M (2016). Functional disorders: children and adolescents. Gastroenterology.

[5] Soares RL (2014). Irritable bowel syndrome: a clinical review. World J Gastroenterol.

[6] Yu SJ, Lee HS, Gung HJ (2022). Efficacy of a restrictive diet in irritable bowel syndrome: a systematic review and network meta-analysis. Korean J Gastroenterol.

[7] Ng QX, Soh AY, Loke W, Lim DY, Yeo WS (2018). The role of inflammation in irritable bowel syndrome (IBS). J Inflamm Res.

[8] Camilleri M, Di Lorenzo C (2012). Brain-gut axis: from basic understanding to treatment of IBS and related disorders. J Pediatr Gastroenterol Nutr.

[9] Rowland I, Gibson G, Heinken A, Scott K, Swann J, Thiele I, Tuohy K (2018). Gut microbiota functions: metabolism of nutrients and other food components. Eur J Nutr.

[10] Gill PA, Inniss S, Kumagai T, Rahman FZ, Smith AM (2022). The role of diet and gut microbiota in regulating gastrointestinal and inflammatory disease. Front Immunol.

[11] Chogle A, Mintjens S, Saps M (2014). Pediatric IBS: an overview on pathophysiology, diagnosis and treatment. Pediatr Ann.

[12] Algera J, Lövdahl J, Sjölund J, Tornkvist NT, Törnblom H (2023). Managing pain in irritable bowel syndrome: current perspectives and best practice. Expert Rev Gastroenterol Hepatol.

[13] Jing F, Zhang J (2014). Metabolic kinetics of 5-hydroxytryptamine and the research targets of functional gastrointestinal disorders. Dig Dis Sci.

[14] Chen M, Ruan G, Chen L (2022). Neurotransmitter and intestinal interactions: focus on the microbiota-gut-brain axis in irritable bowel syndrome. Front Endocrinol (Lausanne).

[15] Fu R, Chen M, Chen Y, Mao G, Liu S (2019). Expression and clinical significance of 5-HT and 5-HT(3)R in the intestinal mucosa of patient with diarrhea-type irritable bowel syndrome. Exp Ther Med.

[16] Stasi C, Bellini M, Gambaccini D (2017). Neuroendocrine dysregulation in irritable bowel syndrome patients: a pilot study. J Neurogastroenterol Motil.

[17] Camilleri M (2012). Peripheral mechanisms in irritable bowel syndrome. N Engl J Med.

[18] Jones J, Boorman J, Cann P (2000). British Society of Gastroenterology guidelines for the management of the irritable bowel syndrome. Gut.

[19] Kridler J, Kamat D (2016). Irritable bowel syndrome: a review for general pediatricians. Pediatr Ann.

[20] Tibble JA, Sigthorsson G, Foster R, Forgacs I, Bjarnason I (2002). Use of surrogate markers of inflammation and Rome criteria to distinguish organic from nonorganic intestinal disease. Gastroenterology.

[21] Thompson W, Longstreth G, Drossman D, Heaton K, Irvine E, Muller-Lissner S (1999). Functional bowel disorders and functional abdominal pain. Gut.

[22] Ford AC, Bercik P, Morgan DG, Bolino C, Pintos-Sanchez MI, Moayyedi P (2013). Validation of the Rome III criteria for the diagnosis of irritable bowel syndrome in secondary care. Gastroenterology.

[23] Palsson OS, Whitehead WE, van Tilburg MA (2016). Rome IV diagnostic questionnaires and tables for investigators and clinicians. Gastroenterology.

[24] Menon J, Thapa BR, Kumari R, Puttaiah Kadyada S, Rana S, Lal SB (2023). Efficacy of oral psyllium in pediatric irritable bowel syndrome: a double-blind randomized control trial. J Pediatr Gastroenterol Nutr.

[25] Thomis M, Claessens AL, Lefevre J, Philippaerts R, Beunen GP, Malina RM (2005). Adolescent growth spurts in female gymnasts. J Pediatr.

[26] Shulman RJ, Hollister EB, Cain K (2017). Psyllium fiber reduces abdominal pain in children with irritable bowel syndrome in a randomized, double-blind trial. Clin Gastroenterol Hepatol.

[27] Guandalini S, Magazzù G, Chiaro A (2010). VSL#3 improves symptoms in children with irritable bowel syndrome: a multicenter, randomized, placebo-controlled, double-blind, crossover study. J Pediatr Gastroenterol Nutr.

[28] Sudha MR, Jayanthi N, Aasin M, Dhanashri RD, Anirudh T (2018). Efficacy of Bacillus coagulans Unique IS2 in treatment of irritable bowel syndrome in children: a double blind, randomised placebo controlled study. Benef Microbes.

[29] Francavilla R, Miniello V, Magistà AM (2010). A randomized controlled trial of Lactobacillus GG in children with functional abdominal pain. Pediatrics.

[30] Karabulut GS, Beşer OF, Erginöz E, Kutlu T, Cokuğraş FÇ, Erkan T (2013). The incidence of irritable bowel syndrome in children using the Rome III criteria and the effect of trimebutine treatment. J Neurogastroenterol Motil.

[31] Ulbricht C, Costa D, M Grimes Serrano J, Guilford J, Isaac R, Seamon E, Varghese M (2010). An evidence-based systematic review of spearmint by the natural standard research collaboration. J Diet Suppl.

[32] Grigoleit HG, Grigoleit P (2005). Gastrointestinal clinical pharmacology of peppermint oil. Phytomedicine.

[33] Micklefield GH, Greving I, May B (2000). Effects of peppermint oil and caraway oil on gastroduodenal motility. Phytother Res.

[34] Narang M, Shah D, Akhtar H (2015). Efficacy and safety of drotaverine hydrochloride in children with recurrent abdominal pain: a randomized placebo controlled trial. Indian Pediatr.

[35] Pesce M, Puoti MG, Rybak A, Andreozzi M, Bruzzese E, Sarnelli G, Borrelli O (2022). Pharmacological interventions for pediatric irritable bowel syndrome. Expert Opin Pharmacother.

[36] Trinkley KE, Nahata MC (2014). Medication management of irritable bowel syndrome. Digestion.

[37] Roohafza H, Pourmoghaddas Z, Saneian H, Gholamrezaei A (2014). Citalopram for pediatric functional abdominal pain: a randomized, placebo-controlled trial. Neurogastroenterol Motil.

[38] Seetharaman J, Poddar U, Yachha SK, Srivastava A, Sen Sarma M (2022). Efficacy of amitriptyline in pediatric functional abdominal pain disorders: a randomized placebo-controlled trial. J Gastroenterol Hepatol.

[39] Saps M, Youssef N, Miranda A, Nurko S, Hyman P, Cocjin J, Di Lorenzo C (2009). Multicenter, randomized, placebo-controlled trial of amitriptyline in children with functional gastrointestinal disorders. Gastroenterology.

[40] Bahar RJ, Collins BS, Steinmetz B, Ament ME (2008). Double-blind placebo-controlled trial of amitriptyline for the treatment of irritable bowel syndrome in adolescents. J Pediatr.

[41] Enck P, Aziz Q, Barbara G (2016). Irritable bowel syndrome. Nat Rev Dis Primers.

[42] Coffin B, Farmachidi JP, Rueegg P, Bastie A, Bouhassira D (2003). Tegaserod, a 5-HT4 receptor partial agonist, decreases sensitivity to rectal distension in healthy subjects. Aliment Pharmacol Ther.

[43] Liem O, Mousa HM, Benninga MA, Di Lorenzo C (2008). Tegaserod use in children: a single-center experience. J Pediatr Gastroenterol Nutr.

[44] Valenzuela JE, Liu DP (1982). The effect of variations in intragastric pressure and gastric emptying of a saline meal in humans. Scand J Gastroenterol.

[45] Karunanayake A, Devanarayana NM, de Silva A, Gunawardena S, Rajindrajith S (2018). Randomized controlled clinical trial on value of domperidone in functional abdominal pain in children. J Pediatr Gastroenterol Nutr.

